# Hydrogen cyanamide breaks grapevine bud dormancy in the summer through transient activation of gene expression and accumulation of reactive oxygen and nitrogen species

**DOI:** 10.1186/s12870-016-0889-y

**Published:** 2016-09-15

**Authors:** Boonyawat Sudawan, Chih-Sheng Chang, Hsiu-fung Chao, Maurice S. B. Ku, Yung-fu Yen

**Affiliations:** 1 Ph.D. Program of Agricultural Science, National Chiayi University, Chiayi, 60004 Taiwan; 2 Department of Farmers’ Services, Council of Agriculture, Taipei, 10014 Taiwan; 3 Tainan District Agricultural Research and Extension Station, Tainan, 71246 Taiwan; 4 Department of Bioagricultural Sciences, National Chiayi University, Chiayi, 60004 Taiwan; 5 School of Biological Sciences, Washington State University, Pullman, WA 99164-4236 USA

**Keywords:** Hydrogen cyanamide, ROS, Dormancy breaking buds, Grapevines, Gene ontology

## Abstract

**Background:**

Hydrogen cyanamide (HC) and pruning (P) have frequently been used to break dormancy in grapevine floral buds. However, the exact underlying mechanism remains elusive. This study aimed to address the early mode of action of these treatments on accumulation of reactive oxygen species (ROS) and reactive nitrogen species (RNS) and expression of related genes in the dormancy breaking buds of grapevine in the summer.

**Results:**

The budbreak rates induced by pruning (P), hydrogen cyanamide (HC), pruning plus hydrogen cyanamide (PHC) and water (control) after 8 days were 33, 53, 95, and 0 %, respectively. Clearly, HC was more effective in stimulating grapevine budbreak and P further enhanced its potency. In situ staining of longitudinal bud sections after 12 h of treatments detected high levels of ROS and nitric oxide (NO) accumulated in the buds treated with PHC, compared with HC or P alone. The amounts of ROS and NO accumulated were highly correlated with the rates of budbreak among these treatments, highlighting the importance of a rapid, transient accumulation of sublethal levels of ROS and RNS in dormancy breaking. Microarray analysis revealed specific alterations in gene expression in dormancy breaking buds induced by P, HC and PHC after 24 h of treatment. Relative to control, PHC altered the expression of the largest number of genes, while P affected the expression of the least number of genes. PHC also exerted a greater intensity in transcriptional activation of these genes. Gene ontology (GO) analysis suggests that alteration in expression of ROS related genes is the major factor responsible for budbreak. qRT-PCR analysis revealed the transient expression dynamics of 12 specific genes related to ROS generation and scavenge during the 48 h treatment with PHC.

**Conclusion:**

Our results suggest that rapid accumulation of ROS and NO at early stage is important for dormancy release in grapevine in the summer, and the identification of the commonly expressed specific genes among the treatments allowed the construction of the signal transduction pathway related to ROS/RNS metabolism during dormancy release. The rapid accumulation of a sublethal level of ROS/RNS subsequently induces cell wall loosening and expansion for bud sprouting.

**Electronic supplementary material:**

The online version of this article (doi:10.1186/s12870-016-0889-y) contains supplementary material, which is available to authorized users.

## Background

Grapevine is a perennial crop in temperate climates and perceives short day petoperiod and cool temperatures as signals to onset bud endodromancy to survive winter conditions; subsequently it requires exposure to adequate chilling temperatures for several weeks (or months) to end the dormancy [1–3]. Endodormancy is regulated by the physiological factors inside the affected buds [3, 4]. Abascisic acid (ABA) accumulates during the development of endodormancy in grapevine by suppression of bud meristem activity and its degradation is critical for dormancy release [5]. Furthermore, ethylene is suggested to participate in the degradation of ABA and budbreak by modulating the expression of ABA signaling regulators [5, 6]. Grapevine grown in subtropical regions often exhibits non-uniform or delayed budbreak in early spring due to warm winter that provides inadequate chilling. This represents a major obstacle for the commercial production of table grapes. Hydrogen cyanamide (H_2_CN_2_, HC) has been found very effective for breaking the dormancy of floral buds in grapevine [7–9], kiwifruit [10] and apple [11, 12]. In subtropical regions, HC treatment on intact dormant buds in spring is an important grapevine orchard management practice to induce uniform budbreak. In Taiwan, a subtropical region, this treatment is also a common practice in mid-summer for the second grapevine harvest in the winter, where paradormant buds on the pruned canes can be treated with HC to ensure effective budbreak. Paradormancy in the latent buds in the summer is regulated by plant growth regulators originating from other organs, such as auxin from the apical meristem, affecting apical dormancy [3, 4]. Few studies have been focused on the mechanism of paradormancy and its release by HC. An understanding of the biochemical and molecular bases underlying the release of dormancy by HC is critical for commercial production of grapes in subtropical and tropical regions.

HC is commonly used as a nitrogen fertilizer with herbicidal and fungicidal effects. It is readily taken up by plant tissues and rapidly decomposed by cyanamide hydratase to urea, followed by urease to ammonium [13]. Rapid detoxification of the highly toxic ammonia is achieved by the GOGAT pathway to produce arginine, histadine and lysine. However, cyanamide is also a very reactive substance and belongs to the classic nitriles. Nitrile hydratases metabolize nitriles to the corresponding amides glutamine and asparagine. However, at high concentrations it is toxic to plants when the enzymatic breakdown of cyanamide exceeds the ammonia detoxification capacity. Cyanamide is well known for its effect to break dormant buds of fruit trees. The most dramatic physiological effect of cyanamide to plants is its strong inhibition of catalase, caused by the reaction of the nitrile group with the thiols and haematin of the enzyme, and the subsequent increase in H_2_O_2_ content [13]. After HC application, cyanide (CN) is released and breaks dormant flower buds in *Prunus* species [14]. Cyanide is also a co-product of cyanogenic glucoside hydrolysis [6, 15, 16]. Cyanide is toxic to plants by arresting aerobic respiration (e.g. cellular hypoxia) and energy production. Consequently, a shift to anaerobic respiration is induced.

Plants respond to HC or potassium cyanide (KCN, respiration inhibitor) by eliciting the reactive oxygen species (ROS) such as H_2_O_2_, as shown in sunflower seeds [15, 17] and in grapevine buds [15, 18]. Hypoxia (8 % O_2_), and two inhibitors of respiration (e.g. KCN and sodium nitroprusside) also triggers the production of H_2_O_2_ and ethylene, which in turn activates the antioxidant systems in grapevine buds through the mediation of the these signaling molecules [19]. Consistently, during germination the seeds develop an anaerobic condition after imbibition due to the rapid consumption of O_2_ and the barrier imposed by the seed coat for gas exchanges, and the depletion of O_2_ in seeds is accompanied by an increase in H_2_O_2_ and NO levels [20]. Taken together, these results suggest that hypoxia maybe the primary cause that induces budbreak and the increased levels of ROS and NO are the secondary products produced in response to hypoxia. A general model accounting for the major events occurring during artificially induced bud dormancy release has been proposed [5, 7, 19, 21]. In this model, upon HC treatment a respiratory disturbance in mitochondria leads to a transient oxidative stress expressed as an increased level of ROS, decreased activity of TCA cycle and decreased production of ATP and increased production of ethylene. To cope with energy crisis, alternative respiratory pathway, glycolysis, pyruvate metabolism and anaerobic respiration or fermentation is induced. In parallel, various antioxidant systems are upregulated to cope with the transient oxidative burst. However, the underlying mechanism leading to growth resumption remains elusive.

ROS are known to play a key role in cell wall loosening in growing tissues [22, 23] and act as signalling molecules in signal transduction in cells, regulating plant growth and development in response to biotic and abiotic stimuli [24, 25]. In plant cells, ROS production is regulated spatially and temporally from many sources and ROS reactivities take place in various cellular components, such as chloroplast, mitochondria, peroxisome, endoplasmic reticulum, apoplast, plasma membrane and cell wall [24, 26–28], with mitochondria as the major source of ROS production [29, 30]. Accumulation of endogenous ROS in plants can be triggered by many environmental stresses, such as water deficit and salinity [31] and chilling stress [32], especially under high light or in combination with other stresses. Generation of ROS can be catalysed by many enzymes, such as glucose oxidase, xanthine oxidase, peroxidases, oxalate oxidases, amine oxidase, lipoxygenases, quinine reductases and NADPH oxidases [24, 33].

Membrane-bound NADPH oxidases, known as respiratory burst oxidase homologues (RBOHs), serve as important molecular ‘hubs’ during ROS mediated signalling in plants [33]. NADPH oxidases control plant growth and development by making ROS that regulate plant cell expansion through the activation of Ca^2+^ [34], integrating calcium signalling and protein phosphorylation with increasing ROS production [25]. Also, the bioreactive lipoxygenase (LOX) metabolites stimulate the activity of NADPH oxidases and production of ROS [35]. The major bulk of lipoxygenases (LOXs) is localized in the cytoplasm and vacuole of the plant cell [36]. Expression of *LOX* is regulated by different forms of stress, such as wounding, water deficiency [37, 38], or pathogen attack [39]. Thus, NADPH oxidases have a dedicated function of generating ROS and act as key signalling nodes integrating multiple signal transduction pathways in plants [40, 41].

ROS in the form of H_2_O_2_ is moderately reactive and relatively long-lived that can pass freely through membranes by diffusion and acts as a messenger in the stress signalling response [42, 43]. H_2_O_2_ upregulates transcription factors (TFs) and TF-interacting proteins, affecting cell division, stem branching, flowering time and flower development [44]. The gaseous nitrogen reactive species (NRS) NO may serve as an enhancer in the ROS generation network [45, 46]. As a key signalling molecule, NO functions in different intercellular processes, including the expression of defense-related genes against pathogens and apoptosis/program cell death (PCD), maturation and senescence, stomatal closure, dormancy release during seed germination, root development and induction of ethylene emission. Recent studies showed that NO can be produced in plants by enzymatic and non-enzymetic systems. The major NO-producing enzymes in plants are nitrate reductase in a NADH-dependent reaction and several arginine-dependent nitric oxide synthase-like (NOS) activities in different cellular compartments [20, 47]. Other potential enzymatic sources of NO include NO synthase, xanthine oxidoreductase, peroxidase, cytochrome P450, and some hemeproteins.

To control ROS levels under oxidative stress, organisms induce a variety of antioxidant enzymes and compounds to scavenge ROS and RNS in the cells. Within a cell, the superoxide dismutases (SODs) in various cellular organelles constitute the first line of defense against ROS [48]. Other defense enzymes, including catalase (CAT), ascorbate peroxidase (APX), guaiacol peroxidase (GPX), glutathione reductase (GR), monodehydroascorbate reductase (MDHAR), and dehydroascorbate reductase (DHAR), protect their cellular constituents by scavenging the harmful ROS and thus maintaining the normal cellular redox state [49]. The antioxidant compounds ascorbate and glutathione serve as cofactors in some of these scavenging reactions. Earlier studies showed that HC inhibits grapevine bud catalase gene expression during the first 4 days of treatment, but induces transcripts for the enzymes pyruvate decarboxylase (*PDC*) and alcohol dehydrogenase (*ADH*) [50]. In grapevine buds, HC also upregulates oxidative stress-related genes, such as thioredoxin h (*Trxh*), glutathione S-transferase (*GST*), ascorbate peroxidase (*APX*), glutathione reductase (*GR*), and hypoxia related genes, such as sucrose synthase (*SuSy*) [51, 52]. After exposure to HC, peroxidase activity in a number of plants is increased. Peroxidases utilize different organic electron donors to reduce H_2_O_2_. Natural chilling also leads to similar induction of these genes during the last stage of the dormancy cycle of grape buds [53]. Class 1 nonsymbiotic hemoglobin is involved in scavenging of NO [20, 54–57]. Its expression is increased during hypoxic stress, application of respiratory chain inhibitors (e.g. cyanide) and high level of nitrate.

Accumulation of excessive ROS (e.g. H_2_O_2_) and RNS (e.g., NO) in turn induces the activation of alternative electron-transport pathway to prevent accumulation of excessive ROS, and the expression of alternative oxidase gene (*AOX1a*) is also known to respond to various stresses in plants [58]. The expression of *AOX1* affects both ROS and RNS generation and accumulation through the respiratory chain in mitochondria [59–61]. Expression of *AOX* is up-regulated in grapevine buds by HC treatment [7], but HC treatment increased H_2_O_2_ production in grapevine buds [18]. Clearly, a complicate regulatory network controlling ROS metabolism exists in plant cells. Moreover, as many genes exist in a gene family whether all members in the family are affected by the same stimuli is not known. Thus, how HC exactly affects the ROS regulatory network during grapevine dormancy release remains unclear.

To gain a better understanding of the underlying mechanism of the release of paradormancy in the summer grapevine buds by P and HC, this study aimed to follow the changes of endogenous ROS and NO levels in the intact dormancy breaking buds by cytochemical staining and identify the candidate genes being significantly altered by microarray analysis. GO categories that are related to ROS-generating, ROS-scavenging, and NO detoxification were identified as key early factors in grapevine dormant bud break. The expression dynamics of these factors during the early stages of budbreak support the importance of a rapid accumulation of ROS and RNS with a concomitant activation of related genes for budbreak.

## Results

### Budbreak rate

**Fig. 1:**
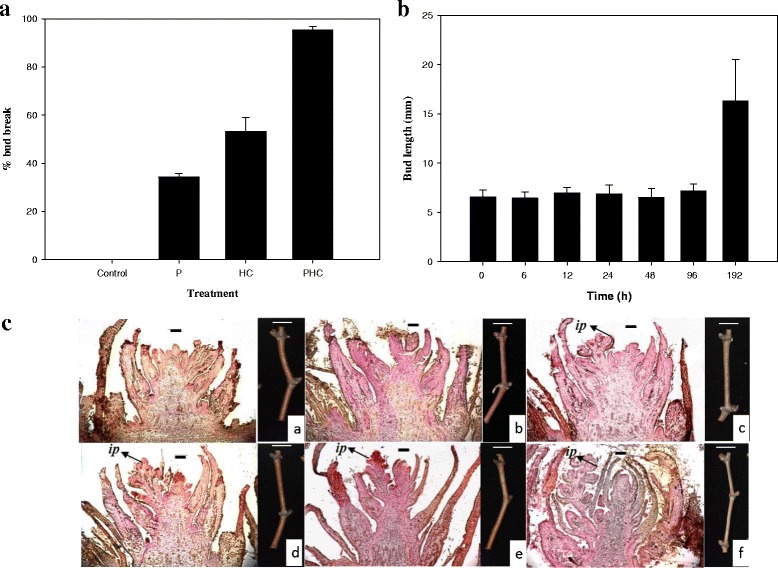
Morphological and anatomical changes in grapevine buds during dormancy break. Percentages of budbreak after 192 h of treatment with P, HC, PHC, or water as a control (*n* = 10, bar: standard deviation) (**A**). Changes in bud length after PHC treatment at 0, 6, 12, 24, 48, 96 and 192 h (*n* = 10, bar: standard error) (**B**). Longitudinal sections of grapevine floral bud development during release of dormancy after PHC treatment: (a) 0 h, (b) 6 h, (c) 12 h, (d) 24 h, (e) 48 h and (f) 96 h. *ip*: inflorescence primordium (**C**). Bar: 200 μm

After 8 days, the budbreak rates of dormant grapevine buds treated with P (pruning), HC (1 % or 12.5 mM hydrogen cyanamide), PHC (P + HC) and water (as a control) were 33.3, 53.3, 95.3 and 0 %, respectively (Fig. 1a). Obviously, HC alone was more effective in promoting dormant budbreak than P. The effect of HC on breaking dormant buds was further enhanced by P. A combined P and HC (PHC) treatment gave rise to the highest dormant budbreak, showing a synergetic interaction between P and HC. During the treatment with PHC, the breaking buds enlarged and sprouted rapidly between 96 and 192 h (Fig. 1b). Anatomical examination before and after PHC treatment showed an active growth resumption of floral meristem starting at 12 h (Fig. 1C-c). Four to five leaf primordia were visible at 12 h and the inflorescence meristem began to develop at 48 h (Fig. 1C-e), and the inflorescence primodia became well developed with complete floral meristem at 96 h post treatment (Fig. 1C-f).

### In situ detection of H_2_O_2,_ O_2_^•-^ and NO

**Fig. 2:**
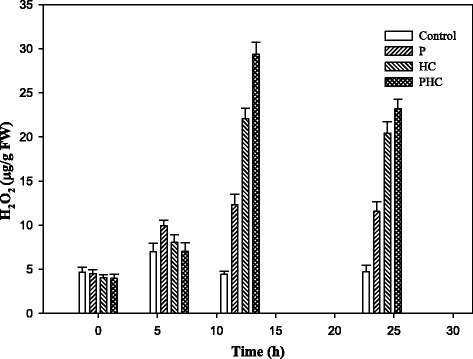
Levels of H_2_O_2_ in the control, P, HC and PHC treated buds. Hydroperoxide was assayed by the ferric-xylenol orange (Fe-XO) complex [62]. After incubation in the dark for 30 min, the absorbance was read at 560 nm with 100 μM xylenol orange as blank

**Fig. 3:**
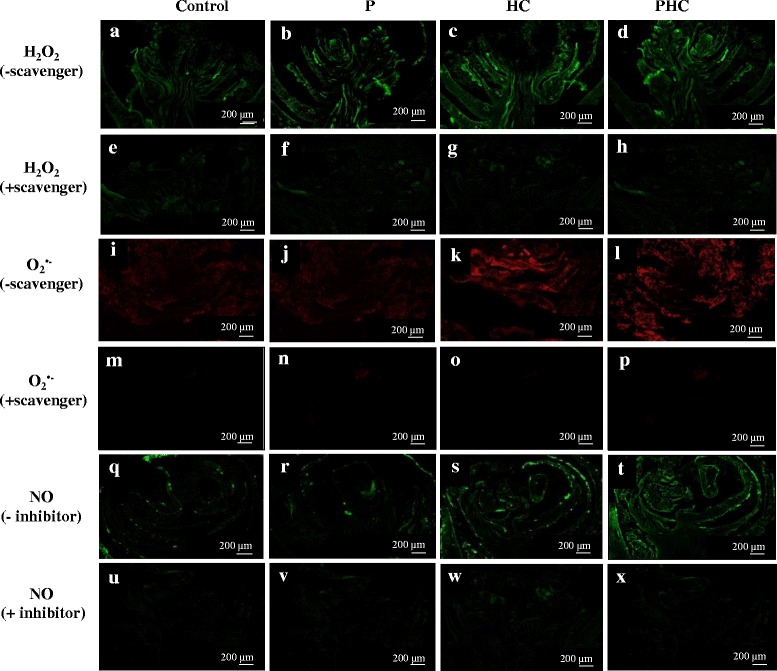
In situ detection of H_2_O_2,_ O_2_^•-^ and NO at early stages of grapevine budbreak. Visualization of H_2_O_2_ by fluorescence microscopy using DCF-DA assay in grapevine bud sections after 12 h of treatment: H_2_O as a control (**a**), P (**b**), HC (**c**) and PHC (**d**). For negative control, grapevine bud sections were incubated with 1 mM sodium pyruvate, an H_2_O_2_ scavenger: control (**e**), P (**f**), HC (**g**) and PHC (**h**). Visualization of O_2_^•-^ by reaction with 10 μM dihydroethidium (DHE) in grapevine bud sections: control (**i**), P (**j**), HC (**k**) and PHC (**l**). For negative control, grapevine bud sections were incubated in 1 mM tetramethylpiperdinooxy, an O_2_^•-^ scavenger: control (**m**), P (**n**), HC (**o**) and PHC (**p**). Visualization of nitric oxide (NO) by DAF-2DA assay in grapevine bud sections: control (**q**), P (**r**), HC (**s**) and PHC (**t**). For negative control, grapevine bud sections were incubated in 10 μM carboxy-PTIO, an NO inhibitor: control (**u**), P (**v**), HC (**w**) and PHC (**x**). Bar: 200 μm

The accumulation of H_2_O_2_ in the grapevine buds during the treatments was first quantitatively determined by measuring the Fe-H_2_O_2_ complex in the buds [62]. For all four treatments, H_2_O_2_ increased rapidly and almost linearly, reaching its maximum level after 12 h and decreased slightly thereafter (Fig. 2), and the averaged amounts on a fresh weight basis were 1.38, 12.24, 22.06, and 29.31 μg/g for the control, P, HC, and PHC treatments, respectively. The accumulation of H_2_O_2_, O_2_^•-^ and NO was subsequently examined by staining in situ in the bud longitudinal sections after 12 h of treatment. A similar pattern of accumulation among these treatments for H_2_O_2,_ O_2_^•-^ and NO was also observed, with the highest levels detected in the PHC treated buds and the lowest levels in the control buds. When the sections were incubated with specific scavengers of ROS or inhibitor of NO, no staining occurred, showing a low background as in the control sections. The results confirmed the specificity of the in situ staining of these reactive species. Thus, H_2_O_2,_ O_2_^•-^ and NO significantly accumulated in the sections of buds treated with PHC, followed by HC and P (Fig. 3). The results suggest that intensive H_2_O_2,_ O_2_^•-^ and NO production took place in the treated dormant buds at the early stage of budbreak. These levels are positively correlated with the percentages of budbreak (Fig. 1a).

### Microarray analysis of differentially expressed genes (DEGs) and clustering of expressed genes

To gain insights into the early mode of molecular action during grapevine budbreak transcriptomic profiles in buds treated with P, HC and PHC for 24 h were examined by microarray using Agilent 44 K Gene Expression Array with specific probes for grapevine genes. An earlier time course study showed that the numbers of up- and down-regulated genes in grapevine buds treated with HC reached the peak around 24 h post-treatment [7]. Clustering of the expressed genes into functional categories in the buds treated with P, HC, and PHC were based on GenSpring analysis, and the changes in transcript abundance were identified by M/A plot. A larger distribution of transcript abundance was found in the buds treated with PHC, followed by HC and P (Additional file 1), consistent with its strong potency on dormancy release. However, it must be pointed out that transcriptional activation of some genes that occurred within 24 h of treatment may have not been identified.

### Identification of common differentially expressed genes (DEGs) among treatments

**Fig. 4:**
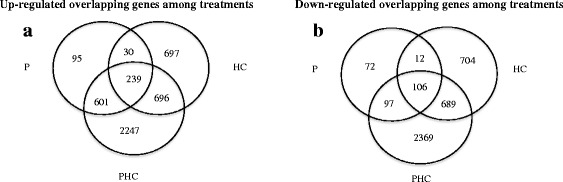
Venn diagrams to show the overlap of significantly up- and down-regulated genes among different treatments. Comparison of significantly up- (**a**) and down–regulated genes (**b**) among P, HC and PHC treatments after 24 h of treatment. Microarray data analyses yielded 6410 significantly up- and 5059 significantly down-regulated genes by the 3 treatments with at least two fold changes (*p* < 0.05, *n* = 3)

Analysis by GenSpring indicated that a total of 965, 1662 and 3783 genes showed significantly up-regulated expression and a total of 287, 1511 and 3261 genes showed significantly down-regulated expression by P, HC and PHC treatments, respectively (Fig. 4a, b). Clearly, alteration in expression of many genes is involved in dormant budbreak in grapevine and many more genes were up-regulated than down-regulated by these treatments, similar to that reported in grape after treatment with HC [7]. 239 up-regulated and 106 down-regulated genes were common for all three treatments, indicating similar molecular events occurred in these buds (Additional file 2). Among the commonly up-regulated genes with a greater than a log2-fold change in expression are those coding for TF factors (e.g. 7 bHLHs, 2 WRKYs, EGL, ERF073, NAM-B1, OFP5 and HEC2) and functional genes coding for protein kinase, peroxidase, ion oxygenase, amine oxidase, PR proteins, dirigent proteins, expansin and extension (Table 1). The expression of some genes related to degradation of cyanamide (e.g. bifunctional nitrilase and nitrile hydratase), synthesis of pigments (e.g. stilbene, anthocyanidin) and metabolism of growth regulators (e.g. ethylene, salicylic acid, brassinosteroid, auxin and cytokinin) was also upregulated. In particular, the hypoxia related gene coding for sucrose synthase (*SuSy*) was significantly up-regulated by all three treatments, especially by PHC. Among the commonly down-regulated genes are those coding for TFs (e.g. ERF016, ERF010-like, DET010-like, NAC18, NAC29, NAC100, FUS3, MYB24, A-7a-like, ABI5, GATA 24-like, ATHB-2, ATHB40) and functional genes coding for chloroplast chaperone danJ11, PR proteins, protein kinase, ubiquitin ligase, cytochrome P-450 and peroxidase 10 and 25. These genes are generally related to stress responses in hormonal action, ROS metabolism, and transition to growth resumption process.

**Table 1:** Functions of important transcription factors and proteins

TFs or proteins	Function	References
Up-regulated		
1. bHLH	bHLHs have a range of different roles in plant cell and tissue development as well as plant metabolism.	[68]
2. WRKY	Regulators involved in various develop006Dental and physiological process, especially in coping with diverse biotic (e.g. wounding, salicylic acid, cold, and salinity) and abiotic (e.g. methyl jasmonate (MeJA) stresses.	[66, 99]
3. ERF	Plays a crucial role in plant growth and development and in response to biotic and abiotic stress conditions in plant. It’s also involved in fruit ripening and stress processes.	[67]
4. NAM-B1	Acts in tissue specific manner to regulate monocarpic senescence and grain filling, and it’s related to carbohydrate metabolism in stems and the grain, and associated with the grain protein content in Fennoscandian wheat.	[69, 70]
5. Protein kinase	Protein kinases are universal signal transduction modules in eukaryotes, including yeasts, animals and plants.	[73]
6. Peroxidase	Peroxidases as key players during the whole life cycle of a plant, and particularly in cell wall modifications, and in roles that can be antagonistic depending on the developmental stage.	[74]
7. Amine oxidase	Cell wall maturation and lignification during development as well as with wound-healing and cell wall reinforcement during pathogen invasion.	[26]
8. NADPH oxidase	A plasma membrane NADPH oxidase produces ROS in planta or in elicited cells during incompatible interaction	[100]
9. PR proteins	The class 1 pathogenesis-related (PR) proteins are thought to be involved in plant defense responses against infection by pathogens, such as fungi or viruses.	[75, 76]
10. Expansin	Expansins are involved in enhancing growth by mediating cell wall loosening.	[77, 98]
11. Extensin	Extensins are involved in defense and in the control of extension growth by differential expressing under stress and non-stress conditions. Extensin genes are developmentally regulated and induced by wounding, methyl jasmonate, abscisic and salicylic acid.	[78–80]
Down-regulated		
1. NAC	NAC has a variety of important functions in plant development, and also in abiotic stress responses.	[101]
2. FUS3	A positive regulator of seed responses to ABA and mediates osmotic stress responses during seed development.	[81, 102]
3. MYB	Regulation of anthocyanin biosynthesis in the grape via expression of the UFGT gene (UDP glucose: flavonoid -3-O-glucosyltransferase).	[85, 103]
4. ABI5	Insensitive to ABA in seed germination, with an earlier flowering phenotype.	[82]
5. GATA-like	GATA DNA motifs have been implicated in light-dependent and nitrate-dependent control of transcription.	[86]
6. ATHB	ATHB10 regulates root hair development, ATHB8 promotes vascular cell differentiation and positively modulates the activity of procambial and cambial cells to differentiate, ATHB2 and ATHB4 genes are strongly induced by far-red-rich light.	[83, 84, 104]
7. Chaperone dnaJ	Regulation of the heat shock response by serving as an important pathway for the folding of newly synthesized polypeptides.	[87, 88]
8. Ubiquitin ligase	Ubiquitin ligase is an important part of cellular regulation in Arabidopsis, suggesting a major role for protein degradation in control of plant life.	[89, 105]

### Classification of up- and down- regulated genes involved in ROS/NO metabolism

GO categories by ErmineJ analysis of ROS related genes that were up- or down-regulated in molecular function 24 h post-treatment were listed in Table 2. Many ROS- and NO-related genes showed significant alteration in expression. We identified clear overlaps of the molecular functions among the up- and down-regulated genes in response to P, HC and PHC treatments. GO terms in which their gene expression levels altered significantly (by at least two fold) by the treatments were listed in Additional file 3. PHC induced more up- and down-regulated genes than HC and P treatments. Also, there are genes commonly regulated by all three treatments. Among the ROS-generating genes, the two respiratory burst oxidase homolog genes related to GO:0043167 (ion binding), *VvRBOHE* and *VvRBOHA,* were down-regulated by PHC, while only *VvRBOHA* was down-regulated by HC. However, *VvRBOHE* was upregulated by P and HC.

**Table 2:** Number of genes that were expressed in each treatment, as identified by GO categories

ROS-/NO-related genes	GO category	Gene family	No. of gene up/down regulated		
			P	HC	PHC
ROS-related genes					
ROS-generating genes	GO:0043167 ion binding	Respiratory burst oxidase homolog protein (VvRBOH)	0/0	0/1	0/2
	GO:0004601 peroxidase activity	Peroxidase (VvPOD)	10/1	10/14	15/10
ROS-scavenging genes	GO:0016209 antioxidant activity	Alpha-dioxygenase 1 (VvDOX1)	1/0	1/0	0/0
		Glutathione peroxidase (VvGPX)	0/0	0/1	1/1
		Catalase isozyme 1-like (VvCAT)	0/0	0/2	0/2
		Cytosolic ascorbate peroxidase (VvAPX)	0/0	0/1	0/2
		Superoxide dismutase [Fe], chloroplastic (VvFSD)	0/0	0/0	1/1
		Alternative oxidase 2, mitochondrial (VvAOX2)	0/0	1/0	1/0
ROS-specific transcription factors	GO:00037000 transcription factor	Heat Stress transcription factor (VvHSF)	0/1	2/2	3/6
		Ethylene response element binding factor (VvERF)	4/3	8/6	8/9
		WRKY transcription factor (VvWRKY)	1/0	9/4	7/7
NO-related gene					
NO-scavenging	GO:0005488 Binding	Hemoglobin-2 (VvHB2)	0/0	1/0	1/0

These were significantly up/down regulated genes related to ROS/NO metabolism in grapevine buds treated with P, HC and PHC following the GO categories by ErmineJ analysis

The ROS-scavenging peroxidases related to GO:0004601 exist in a big gene family, and many of *VvPOD* genes were up-/down-regulated, especially by HC and PHC (10/14, and 15/10). Obviously, most of the *POD* genes were upregulated and they are assumed to play a key role in the reduction of H_2_O_2_ in grapevine buds treated with HC [13]. For other ROS-scavenging genes in GO:0016209 (antioxidant activity), the expressions of five gene families have been up- or down-regulated by the three treatments. For example, one alpha-dioxygenase gene (*VvDOX1*) was up-regulated by P and HC, but not by PHC. Gene coding for glutathione peroxidase *(VvGPX2)* was up-regulated by PHC whereas another (*VvGPX8)* gene was down-regulated by HC and PHC. After 24 h of treatment, two catalase isozyme 1-like genes (*VvCAT*) were down-regulated by both HC and PHC. In addition, *VvAPX* coding for cytosolic ascorbate peroxidase was down-regulated by both HC and PHC, and *VvFSD* coding for chloroplastic superoxide dismutase (SOD) [Fe] was down-regulated by PHC whereas *VvFSD3* was up-regulated by PHC. The expression of *VvAOX* that codes for alternative oxidase in mitochondria was up-regulated by both HC and PHC. For NO related genes, the expression of hemoglobin-2 gene (*VvHB2)* in binding (GO:0005488) was significantly up-regulated by both HC and PHC. The expression of the genes in the three ROS related transcription factor gene families (GO:0003700), namely heat stress transcription factor *(VvHSF),* ethylene response element binding factor *(VvERF)* and WRKY transcription factor (*VvWRKY),* was also differentially altered. The numbers of *VvHSF* genes up-/down-regulated by P, HC, and PHC were 0/1, 2/2, and 3/6, respectively, while the numbers of *VvERF* genes up-/down-regulated expressed by these treatments were 4/3, 8/6, and 8/9, respectively. Many of the *VvWRKY* genes were also up-/down-regulated, especially by HC and PHC (9/4 and 7/7), compared to P (1/0). These results indicate that more genes are altered in their expression at a higher intensity by PHC and the expressions of different genes in the same GO category are differentially regulated by these treatments.

### Expression dynamics of ROS/NO-related genes during PHC treatment

**Fig. 5:**
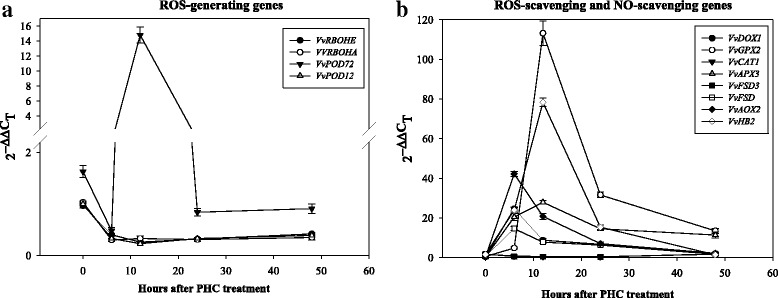
Changes in transcript abundance of 12 up- and down-regulated ROS/NO-related genes during 48 h treatment with PHC, as analyzed by qRT-PCR. These include 4 ROS-generating genes (*VvRBOHE, VvRBOHA, VvPOD72, VvPOD12*) (**a**), and 7 ROS-scavenging genes (*VvDOX1*, *VvGPX*2, *VvCAT1, VvAPX3, VvFSD3, VvFSD, VvAOX2*) and one NO-scavenging gene (*VvHB2*) (**b**). The selection of these genes was based on their relative expression levels obtained by the microarray analysis (Table 3)

Quantitative RT-PCR (qRT-PCR) was used to confirm the alteration in expression of 12 selected DEGs identified by cDNA microarray and profile the expression dynamics of these genes during the 48 h of PHC treatment using *eIF4A* as a reference gene as its expression did not change substantially [1]. The DEGs included 2 genes coding for ROS-generating (*VvRBOHE, VvRBOHA*), 9 genes coding for ROS-scavenging (*VvPOD72,* and *VvPOD12, VvDOX1*, *VvGPX2, VvAPX3, VvFSD3, VvFSD, VvAOX2, VvCAT1*) and one gene coding for NO-scavenging (*VvHB2*) (Table 3). The expression profiles of these 12 genes during the 48 h treatment showed different tempos, with most of their expression peaked at 6 or 12 h, indicating a transient nature. A similar trend in gene expression was also reported in detached grapevine canes treated with HC or heat shock (HS) [7]. Two of the ROS-generating respiratory burst oxidase homologue genes, *VvRBOHE* and *VvRBOHA*, and the scavenging peroxidase gene *VvPOD12* were down-regulated by more than two folds throughout the first 48 h of treatment. In significant contrast, the expression of the peroxidase gene *VvPOD72* started to increase after 6 h of treatment and reached its maximum expression at 12 h (14.8 fold), but dropped off to normal levels thereafter, consistent with the results obtained by microarray (Table 3). Thus, *VvPOD72* and several other members of the peroxidase family (Table 2) played an important role in early ROS metabolism by scavenging H_2_O_2_ upon PHC treatment, which synchronized with the new meristem growth (Fig. 5a).

**Table 3:** List of genes that were used to profile their expression dynamics by qRT-PCR

Gene Locus ID	Gene description	Normalized Fold (Log_2_)		
		P	HC	PHC
*GSVIVT00016386001*	Vitis vinifera respiratory burst oxidase homolog protein E (VvRBOHE)	0.30 ± 0.21	1.11 ± 0.18	−1.22 ± 0.06
*GSVIVT00002525001*	Vitis vinifera respiratory burst oxidase homolog protein A (VvRBOHA)	−0.64 ± 0.06	−1.53 ± 0.29	−1.96 ± 0.16
*GSVIVT00031598001*	Vitis vinifera peroxidase 72 (VvPOD72)	1.59 ± 0.77	2.09 ± 1.18	4.19 ± 0.18
*GSVIVT00031723001*	Vitis vinifera peroxidase 12 (VvPOD12)	−0.79 ± 0.05	−0.74 ± 0.25	−1.71 ± 0.13
*GSVIVT00020782001*	Vitis vinifera alpha-dioxygenase 1 (VvDOX1)	1.83 ± 1.16	2.82 ± 0.55	0.13 ± 0.25
*GSVIVT00001920001*	Vitis vinifera glutathione peroxidase 2 (VvGPX2)	0.34 ± 0.39	0.28 ± 0.45	1.10 ± 0.54
*GSVIVT00004080001*	Vitis vinifera catalase isozyme 1-like (VvCAT1)	−0.35 ± 0.17	−1.52 ± 0.02	−1.57 ± 0.15
*GSVIVT00036747001*	Vitis vinifera cytosolic ascorbate peroxidase (VvAPX3)	−0.47 ± 0.24	−1.05 ± 0.09	−1.75 ± 0.04
*GSVIVT00007083001*	Vitis vinifera superoxide dismutase [Fe] 3, chloroplastic (VvFSD3)	0.40 ± 0.17	0.17 ± 0.10	1.82 ± 0.11
*GSVIVT00014163001*	Vitis vinifera superoxide dismutase [Fe], chloroplastic (VvFSD)	0.50 ± 0.13	0.33 ± 0.22	−1.60 ± 0.17
*GSVIVT00003173001*	Vitis vinifera alternative oxidase 2, mitochondrial (VvAOX2)	0.44 ± 0.16	2.43 ± 0.37	1.07 ± 0.18
*GSVIVT00036443001*	Vitis vinifera hemoglobin-2 (VvHB2)	−0.52 ± 0.43	4.41 ± 0.34	3.08 ± 0.43

These include twelve differentially expressed genes related to ROS/NO metabolism in grapevine buds treated with P, HC or PHC for 24 h, as revealed by microarray analysis

For other ROS-scavenging genes, the alpha-dioxygenase *VvDOX1* showed a rapid increase (24.6 fold) in transcript as early as 6 h post treatment and declined gradually throughout the treatment. A small increase (4.8 fold) in the glutathione peroxidase *VvGPX2* transcript was detected as early as 6 h, but its expression accelerated rapidly thereafter and reached its maximum expression (113.2 fold) at 12 h, but dropped off to 31.6 and 13.4 fold at 24 and 48 h, respectively. For the ascorbate peroxidase gene *VvAPX3,* a rapid increase of transcript (20.4 fold) was detected as early as 6 h and reached its maximum expression (27.8 fold) at 12 h and slowly declined to 14.5 and 11.4 fold at 24 and 48 h, respectively. Interestingly, *VvFSD3* and *VvFSD*, both coding for chloroplastic superoxide dismutases (SODs), showed different expression patterns: *VvFSD3* expression decreased progressively from 0.6 fold at 6 h to 0.4 fold at 24 h after treatment but increased to 1.8 folds after 48 h of treatment, whereas the expression of *VvFSD* showed a rapid increase as early as 6 h (14.6 fold) but declined gradually to a normal level at 48 h. The mitochondrial alternative oxidase *VvAOX2* also showed a rapid increase in expression (42.2 fold) as early as 6 h but declined gradually to a normal level at 48 h. It is also noticeable that the expression of the peroxisomal catalase isozyme-like *VvCAT1* was first down-regulated, decreasing progressively from 0.7 fold at 6 h to 0.5 fold at 24 h, but increased to 1.8 fold after 48 h of treatment. The initial downregulation of catalase expression may have contributed to the rapid accumulation of H_2_O_2_ [13], but its subsequent rise in expression may have prevented the buildup of a lethal level of H_2_O_2_. A rapid increase in the hemoglobin-2 (*VvHB2*) transcript was detected at 6 h (23.9 fold) and it reached its maximum expression (78.3 fold) at 12 h, but its transcript level dropped off from 15.33 at 24 h to 1.39 fold at 48 h, similar to that reported in an earlier study [7]. This result suggests that the intensive expression of hemoglobin 2 is triggered by the treatment at very early stage of the treatment and may be important in scavenging NO. Taken together, these results suggest that the two NADPH oxidases, VvRBOHE and VvRBOHA, may not be a key player in ROS generation, while VvPOD72, VvDOX1, VvGPX2, VvAPX3 and VvHB2 are important for ROS and NO scavenging, respectively (Fig. 5b). Overall, these results are in agreement with those obtained by microarray analysis after 24 h of treatment with HC (Table 2, Additional file 3).

## Discussion

HC has been frequently used to break endodormancy of floral buds in grape and several studies have been conducted to address its physiological and molecular basis [4, 8, 9, 13, 63]. However, study on its application to release paradormancy of grape floral buds in the summer has received very little attention. This study aimed to address the early mode of action of PHC, a combination of pruning and HC, with intact grape canes on its effective alleviation of bud dormancy in hot summer that results in uniform budbreak and floral development without chilling requirement. PHC induced budbreak much more rapidly and efficiently than P or HC alone, indicating a synergetic interaction between P and HC treatments (Fig. 1a). Compared to the long period of time required for budbreak by chilling [64], the rapid induction of budbreak by PHC observed in this study may in part be due to the nature of dormancy and the warm temperatures in the summer which accelerate metabolism and growth. Large amounts of H_2_O_2_, O_2_^•-^ and NO accumulated rapidly in the buds upon the treatments, especially by PHC (Fig. 3); and the amount of H_2_O_2_ in the buds increased almost linearly in the buds, reaching its maximum level within 12 h (Fig. 2). Moreover, the ROS levels accumulated are closely correlated with the percentage of budbreak among these treatments, and this strong, transient oxidative burst coincides with growth resumption in the buds (Fig. 1). Similarly, previous studies also showed that grape buds respond to HC and KC by eliciting ROS, such as H_2_O_2_ [18, 19, 65]. Thus, a combination of P and HC is most effective in eliciting rapid accumulation of ROS and release of the paradormant buds in summer grapevine.

To provide insights into the molecular basis of HC effect on breaking dormant buds in grapevine, we conducted transcriptomic analysis during budbreak. Our transcriptomic profile provided a clear link between gene expression and ROS accumulation during the early stage of budbreak. Upon PHC treatment expression of a number of specific genes was altered rapidly to accommodate the metabolic activities required for budbreak and growth resumption. As expected, the numbers of significantly up-/down-regulated genes were highest in PHC treated buds, compared to HC and P treated buds (Fig. 4a, b). Moreover, PHC exerted a higher intensity of regulation, relative to other treatments. Although P, HC and PHC induced dormant budbreak at varying degrees, many transcription factor (TF) and functional genes were commonly induced or suppressed by these treatments (Table 1). These genes/proteins must play important roles in budbreak response. Many of the upregulated TFs, such as WRKYs [66] and ERF (ethylene response factor) [67], are known to be involved in biotic and abiotic stress responses, whereas some other TFs, such as bHLH and NAM-B1, are related to cell and tissue development. For example, bHLHs exhibit a range of different roles in plant cell and tissue development [68], and NAM-B1 acts in a tissue specific manner to regulate monocarpic senescence and grain filling [69, 70]. Consistently, ethylene biosynthesis is reported to increase in grape buds in response to HC and HS treatments and plays a key role in dormancy release by activating ERFs [7], and the high levels of ethylene accumulated in the submerged tissues promote shoot elongation [71] and parenchyma formation [72].

Many of the upregulated functional genes, such as protein kinase, peroxidase, amine oxidase, PR proteins, expansin and extension, are related to plant defense responses and cellular growth. For example, protein kinases are universal signal transduction modules in eukaryotes [73]. Amine oxidase that catalyzes the oxidative de-amination of polyamines and production of H_2_O_2_ has been correlated with cell wall maturation and lignification during development as well as wound-healing and cell wall reinforcement during pathogen invasion [26]. Peroxidases that catalyze the reduction of H_2_O_2_ are known to be involved in cell wall loosening [74]. Thus, these two enzymes may be the key players of ROS metabolism in the cell wall during dormant budbreak in grapevine, as exerted by PHC. PR proteins are thought to be involved in plant defense responses [75] and response to infection by pathogens [76]. In addition, expansin is related to cell wall loosening [77], while extensins expressed under stress and non-stress conditions (e.g. wounding, methyl jasmonate, abscisic acid and salicylic acid) [78, 79] are related to defense and control of extension growth [80]. Thus, these commonly induced TF and functional genes in stress response, oxidative burst, hormonal regulation, and reprogramming of cellular developmental and physiological processes must play important roles in the ROS regulatory network leading to dormancy breaking and bud sprouting in grapevine. Earlier microarray analyses with detached grapevine canes also suggest that HC and HS break the dormant buds through the link between sublethal stress, mitochondrial activity, temporary hypoxic conditions, ethylene metabolism, ROS scavenging activity and cell enlargement during bud dormancy release [7, 52].

Among the down-regulated TFs, NAC is known to be involved in biotic and abiotic stress response, FUS3 controls multiple elementary processes during seed development [81], ABI5 is related to insensitivity to ABA in seed germination and during embryogenesis [82], and ATHB regulates root hair development and promotes procambial and cambial vascular cell differentiation [83, 84]. In addition, MYB regulates proanthocyanidian biosynthesis in grapes [85], and GATA-like has been implicated in light-dependent and nitrate-dependent control of transcription [86]. Among the down-regulated functional genes, chaperone dnaJ regulates heat shock response [87] for proper folding of newly synthesized polypeptides [88] and ubiquitin ligases is an important part of cellular regulation in Arabidopsis [89]. Most of these responses may reflect temporary impairment of normal metabolic activities under the oxidative stress conditions.

### Expression dynamics of ROS metabolism related genes

PHC triggered a rapid generation and accumulation of ROS and NO peaked at 12 h (Figs. 1b, 2 and 3), with a concomitant transcriptional activation of related genes peaked at 6–12 h post-treatment (Figs. 4 and 5). ROS levels are controlled by a network of generating and scavenging enzyme systems. For ROS generation, PHC highly induced *POD72*, but suppressed *RBOHE, RBOHA* and *POD12* at the early stages of treatment. Besides amine oxidase (Table 1), these results emphasize the importance of *POD72* and other peroxidase members in ROS generation in cell wall during paradormant bud break in the summer grapevine. Consistently, previous results also suggest that peroxidase activity is increased largely in grapevine buds upon fulfilment of chilling requirement [64] and it may play an important role in consumption or release of ROS and in cell wall modification [74]. In response to oxidative burst genes coding for the various antioxidant systems, e.g. *GPX2*, *CAT1*, *APX3*, *AOX2*, *DOX1* and *HB2*, were also rapidly turned on in the dormancy breaking buds to scavenge ROS and NO. As reported earlier, after 12–24 h of HC treatment, *GR*, *GST* and *APX* genes are upregulated in grapevine buds [7]. GPX and APX catalyze the reduction of hydrogen peroxide to water by using glutathione and ascorbate, respectively, as a specific electron donor [90]. In addition, glutathione is a strong detoxifying agent and has been shown to bind HC directly on its free thiol groups [91].

In Arabidopsis, α-DOX fatty acid dioxygenase protects oxidative stress and cell death [92]. *AOX1* is known to be induced by H_2_O_2_ and NO [93] and its expression is induced by both HS and HC application in grapevine buds during 96 h of treatment [7] and it may contribute to the reduction of both ROS and RNS generation through the respiratory chain in mitochondria [47]. Therefore, our results are in agreement with the earlier observations that detoxifying mechanisms are correlated with the temporary oxidative stress and H_2_O_2_ may act as a signal in the release of endodormancy in grapevine buds by HC [50]. Taken together, these results support a common mode of ROS regulatory network and its modulation in grapevine dormancy break. For detoxification of RNS, the expression of grapevine HB2 gene peaked at 6 h post-treatment by PHCN (Fig. 5b). Similarly, the expression of HB was markedly up-regulated in the HC treated buds in an earlier study with grape [7]. Besides being an O_2_ carrier, HB serves as an electron carrier during the conversion of NO to NO_3_ in various tissues [20, 54–57]. Therefore, VvHB2 may play a key role in preventing accumulation of excessive NO and its toxicity in grape buds during budbreak.

### Working model integrating physiological and molecular events during paradormancy release by PHC in grapevine buds

Earlier studies suggest a general model accounting for the major events occurring during artificially induced bud dormancy release [4, 5, 7, 13, 19, 21, 45]. Cellular hypoxia is considered the primary cause that induces budbreak while the increased levels of ROS and NO are the secondary products produced in response to hypoxia. Disturbance in mitochondria leads to a transient oxidative stress expressed as an increased level of ROS, decreased activity of TCA cycle and decreased production of ATP and increased production of ethylene. Alternative respiratory pathway, glycolysis, pyruvate metabolism and anaerobic respiration or fermentation is subsequently induced to cope with energy crisis. Our results are in general consistent with this model. However, there are unique features derived from this study that may be attributed to the nature of the plant material used. These studies mainly addressed the release of endodomancy by HC in the spring, while the present study was focused on the release of paradormncy of grapevine buds in the hot summer. For example, ABA is known to accumulate during the development of endodormancy in grapevine by suppression of bud meristem activity and its degradation mediated by ethylene is critical for dormancy release [5]. It is unlikely that well-irrigated grapevines in the summer accumulate ABA in the paradormant buds. In this study, pruning resulted in a budbreak rate of 33 % (Fig. 1a), which is consistent with paradormancy in the latent buds in the summer as regulated by plant growth regulators originating from other organs (e.g., auxin from the apical meristem), affecting apical dormancy [3, 4]. However, the hormone ethylene is clearly involved in growth resumption in both types of dormancy.

**Fig. 6:**
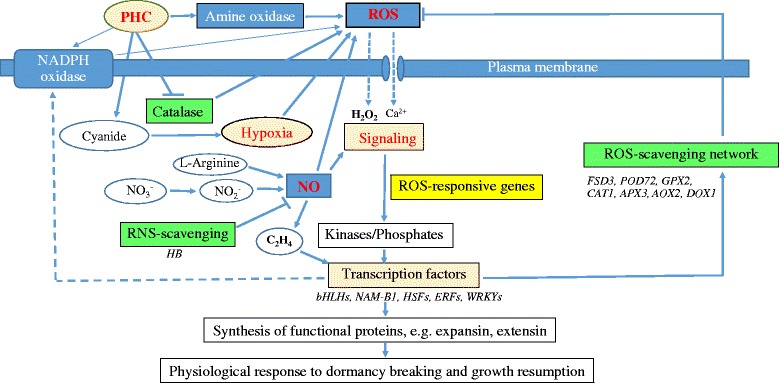
Proposed mechanistic model for paradormancy release by PHC in grapevine buds. Upon application, HC causes immediate cellular hypoxia through the action of released cyanide on aerobic respiration and inhibition of catalase activity and induces H_2_O_2_ production. ROS (O_2_^•-^, H_2_O_2_,^.^OH) and RNS (NO) are rapidly produced possibly by several enzymatic systems, such as NADPH oxidase, amine oxidase, nitrate reductase and arginine-nitric oxide synthase. H_2_O_2_ and ethylene act as signaling molecules activating genes involved in antioxidant defense systems. The antioxidant machinery and related pathways (e.g. FSD, POD, DOX, GPX, APX, AOX, HB, etc.) are upregulated to maintain ROS and RNS at sublethal levels to avoid PCD. H_2_O_2_ in the cell wall induces cell wall loosening and expansion. H_2_O_2_ also functions in anabolic, signaling and defense pathways and in the synthesis of the other ROSs,^.^OH in the presence of H_2_O_2_ and O_2_^•-^ exerts a wall loosening effect, and H_2_O_2_ also drives peroxidase-mediated cross-linking of structural cell wall components. In the signal transduction pathway, ROS triggers the expression of ROS responsive genes. H_2_O_2_ and^.^OH activate Ca^2+^ channels and initiate a signal transduction and activate protein kinase activity, which in turn activates different transcription factors to regulate ROS scavenging and ROS producing pathways. Finally, these transcription factors control the downstream functional gene expression responsible for dormancy release and growth resumption in grapevine buds. The accumulation of ethylene in the buds promotes cell elongation and parenchyma formation by activating related ERFs

Based on our results and the literature, we propose a working model to integrate the early physiological and molecular events underlying the release of paradormancy by PHC in grapevine buds (Fig. 6). Upon PHC treatment, the release of cyanide [6, 14–16] rapidly disturbed aerobic respiration and resulted in cellular hypoxia, and the inhibition of catalase by cyanide promoted H_2_O_2_ accumulation. ROS (O_2_^•-^, H_2_O_2_,^.^OH) and RNS (NO) were also rapidly produced by NADPH oxidase/amine oxidase and NADH dependent nitrate reductase/arginine-nitric oxide synthase, respectively. The gaseous NO in turn served as an enhancer in the ROS regulatory network [46]. To prevent the accumulation of lethal levels of ROS/RNS and PCD, the expression of specific genes involved in various metabolic activities (e.g., AOX, SuSy) and antioxidant systems (e.g., FSD, POD72, APX3, GPX2, CAT1, HB2, and pigments) were induced. H_2_O_2_ and^.^OH activated Ca^2+^ channels and initiated signal transduction through protein kinase activity, leading to expression of specific stress-responsive TFs (e.g., HSFs, WRKYs, ERFs, HLHs, NAM-B1) to regulate ROS producing and scavenging pathways and cellular metabolism [40]. Apoplastic^.^OH in the presence of H_2_O_2_ and O_2_^•-^ interacted with cell wall component leading to modification of cell wall structure for expansion growth [94]. Whereas the ROS produced in the cytosol or transported from the apoplast served as signal molecules [15, 17] and triggered the expression of stress-induced specific TFs for the expression of related downstream functional genes responsible for dormancy breaking and growth resumption (e.g., hormone metabolism, expansin, extension) in grapevine buds. The accumulation of ethylene in the buds promoted cell elongation and chlorenchyma formation by activating related ERFs.

It’s clear that the buildup of a sublethal level of ROS and RNS is a common denominator for bud dormancy release in grapevine in response to natural chilling, HS, P and HC treatments. Enhanced expression of the antioxidant enzymes GST, APX, GR, GPX, AOX and expansin is also reported by HC and HS treatment [7, 51, 52] and during the last stage of chilling requirement in grapevine [53, 64]. Also, genes related to ABA, auxin, ethylene and salicylic acid metabolism are commonly regulated by these treatments. The interplay among these growth regulators leads to cell expansion and division and growth resumption. Thus, judged from the fact that a repertoire of the similar genes is commonly induced by HC, HS, P and natural chilling in a temporary nature, this ROS/NRS regulatory network is essential for the release of bud dormancy in grape and possibly other plants as well.

## Conclusion

In summary, PHC effectively releases paradormancy in grapevine buds in the summer with a rapid accumulation of ROS and RNS and a concomitant transcriptional activation of related genes. Our transcriptional profiling links the rapid transcriptional activation of specific ROS related genes and accumulation of various ROS and RNS underlying the early physiological process during dormant bud break as affected by PHC. The key up-/down-regulated genes identified are all related to ROS metabolism in three important GO categories: peroxidase activity, antioxidant activity and transcription factor. Most of these genes exhibited a peak expression 6–12 h post-treatment, coinciding with the maximum accumulation of ROS at 12 h. The ROS scavenging activities mediated by various antioxidant systems avoid the buildup of lethal levels of ROS and RNS to trigger PCD. The sublethal levels of ROS in turn trigger cell wall loosening and the expression of genes (e.g. expansin, extensin) necessary for cell expansion and extension required for new bud growth as regulated by related hormones (e.g. ethylene). As the overall response to a number of dormancy releasing treatments is remarkably similar, this simplified working model of ROS regulatory network may describe the important molecular and biochemical events during budbreak in grapes and other species as well.

## Methods

### Plant material

The grapevine buds used in this experiment were collected in late August from the mature canes of 5-year-old plants (*V. vinifera.* x *V. labruscana* Bailey cv. Kyoho) grown in the research vineyard of the Taichung District Agricultural Research and Extension Station (Taichung DARES, 24°00’N, 120°53’E, elevation19 M), Changhua County, Taiwan. The day/night temperature in Taichung in Changhua was 27–34 °C/22–27 °C. Plants were fertilized on a regular basis and irrigated with drips on open orchard.

### Dormancy release by different treatments

In late August, uniform grapevine canes (with ca. 25 buds) were identified and divided into three blocks of randomized complete blocks for four treatments: water (as control; CK), pruning (P), 1 % (w/v or 12.5 mM) HC (H_2_CN_2_, 50 % stock, Dormex, SKW, Trostberg, Germany) and a combination of P and HC (PHC). All treatments were applied to the 8^th^ dormant buds from the base of cane. Control buds were sprayed with water until dripping, while buds for HC treatment was sprayed with 1 % (w/v) HC until dripping without pruning. For P treatment, the canes were pruned at 2 cm apart from the 8^th^ node, and for PHC treatment the buds on the 8^th^ node of the pruned canes were sprayed immediately with 1 % (v/v) HC until dripping. After 24 h of treatments, buds on ten single-nodes in each treatment were sampled in 3 replications, frozen immediately in liquid nitrogen and stored at −80 °C until RNA extraction. Freshly harvested buds before and after treatments were also used for microscopic examination, H_2_O_2_ assay, and in situ detection of H_2_O_2,_ O_2_^•-^ and NO. Rates of budbreak were assessed in 30 buds/treatment after 8 days of treatment.

### Microscopic observation of bud development after treatments

Grapevine buds were collected before and after 6, 12, 24, 48 and 96 h of treatments and fixed in FAA solution for 2 h, washed three times with 1X PBS buffer (13.7 mM NaCl, 0.27 mM KCl, 1 mM Na_2_HPO_4_, and 0.18 mM KH_2_PO_4_) and embedded into OCT (optimal cutting temperature) compound (Cryomatrix, Thermo Scientific). Longitudinal sections (10 μm thick) were cut with a microtome (Microm HM550, Thermo Scientific), mounted on glass slides, dehydrated at 60 °C for 1 h, and then stained with filtered 0.1 % Harris Hematoxylin solution (HHS, Sigma) for 10 min. Samples were rinsed in cool running double distilled H_2_O for 5 min, followed by 70 % and 100 % EtOH dehydration before dipping in 0.5 % Erosin (1.5 g dissolved in 300 mL of 95 % EtOH). Finally, the samples were equilibrated in xylene solution before mounting and covering with Leica CV mount. Images of sections were captured with a light microscopy (Axio Imager 2, Carl Zeiss).

### Ferric xylenol orange assay of H_2_O_2_

Frozen grapevine buds (0.10 g) harvested before and during treatments were ground to fine powder in liquid N_2_ and then suspended in 2 mL of 80 % ethanol. After centrifugation, 100 μL supernatant was mixed with 1 mL of reaction solution containing 25 mM H_2_SO_4_, 100 μM xylenol orange, and 250 μM ferrous ammonium sulphate, following the protocol for hydroperoxide assay with the ferric-xylenol orange (Fe-XO) complex [62]. After incubation in the dark for 30 min, the absorbance was read at 560 nm with 100 μM xylenol orange as blank.

### In situ H_2_O_2_, O_2_^•-^ and NO detection by fluorescence microscopy

For in situ staining of H_2_O_2_, O_2_^•-^ and NO, dormant grapevine buds treated with P_,_ HC, PHC and water (as a control) for 12 h were sectioned (10 μm thickness) and stained for H_2_O_2_, O_2_^•-^ and NO with 25 μM 2’, 7’-dichlorofluorescin diacetate (DCF-DA, Calbiochem, San Diego, CA, USA) [95], 10 μM dihydroethidium (DHE, Calbiochem, San Diego, CA, USA) [96], and 10 μM 4,5-diaminonaphthalene (DAF-2DA, Calbiochem, San Diego, CA, USA) prepared in 10 mM Tris-HCl (pH 7.4), respectively. For negative controls, sections were incubated sequentially with 1 mM sodium pyruvate (H_2_O_2_ scavenger), 1 mM tetramethylpiperdinooxy (O_2_^•-^ scavenger), and 1 mM carboxy-PTIO; 2-(4-Carboxyphenyl)-4,4,5,5-tetramethylimidazoline-1-oxyl-3-oxide potassium salt (NO inhibitor), respectively. The bud sections were subsequently washed twice in 1X PBS buffer (13.7 mM NaCl, 0.27 mM KCl, 1 mM Na_2_HPO_4_, and 0.18 mM KH_2_PO_4_) for 15 min each and then embedded in 30 % polyacrylamide blocks. The sections were mounted on glass slides and images captured with a fluorescence microscope (Axio Imager 2, Carl Zeiss).

### RNA extraction and microarray analysis

Frozen grapevine buds were ground to fine powder in liquid N_2_ and RNA was extracted following the protocol of a previous study [97]. Total RNA was purified by RNeasy plant mini columns (Qiagen, Valencia, CA, USA) following the manufacturer’s guide, and RNA quality was verified by 2100 Bioanalyzer RNA 6000 nano chip (Santa Clara, CA. USA). The RINs (RNA Integrity Number) for the RNA samples ranged from 8.6 to 10.0, indicative of low degradation.

### Probes and gene expression microarray

The whole genome mRNA sequences of grape were downloaded in FASTA format from http://www.genoscope.cns.fr/externe/Download/Projets/Projet_ML/data/8X/annotation/Vitis_ vinifera_mRNA v1.fa and saved into a text file. Sequences in text format were downloaded to the eArray web design application from Agilent Technologies (Santa Clara, CA, USA) for probe design. One probe per target was designed in the sense orientation with a 3ʹ bias, using base composition methodology as recommended by the manufacturer for gene expression arrays. The best probes were used for generating a custom microarray arranged in a 4 × 44 K format, using a randomized lay-out. Each array included 30,128 probes (i.e., 30,128 genes, each gene with 1 probe), 60 bp in length, 1264 positive (spike-in) controls and 153 negative control probes distributed across the array. Positive controls were used as a quality control of the cRNA synthesis, labelling and hybridization steps, whereas negative controls were included to estimate fluorescence background and background variance.

### Microarray analyses

Microarray experiment procedures were carried out following the manufacturer’s protocols. Briefly, 1 μg of total RNA was amplified by an Agilent Quick Amp Labeling Kit (Agilent Technologies, USA) and labeled with Cy3-CTP or Cy5-CTP (Agilent Technologies, USA). During the in vitro transcription process the treatment sample RNA was labeled by Cy5 and the control sample RNA was labeled by Cy3. 0.825 μg of Cy-labeled cRNA was fragmented to an average size of about 50–100 nucleotides by incubation with fragmentation buffer (Agilent Technologies, USA) at 60oC for 30 min. Correspondingly fragmented labeled cRNA is then pooled and hybridized to Agilent Whole Grape Genome 4x44K oligo microarray (Agilent Technologies, USA) at 65 °C for 17 h. After washing and drying by nitrogen gun blowing, microarrays were scanned with an Agilent microarray scanner (Agilent Technologies, USA) at 535 nm for Cy3 and 625 nm for Cy5. Scanned images were analyzed by Feature Extraction software 10.5 (Agilent Technologies, USA) to quantify signal and background intensity for each feature, and the data were normalized by rank-consistency-filtering LOWESS method. Finally, the GenSpring (Agilent Technologies, USA) software was used to identify significant differentially expressed genes (DEGs). The raw data is available at the Gene Expression Omnibus (http://www.ncbi.nlm.nih.gov/geo/info/linking.html) under the accession GSE86163.

### Differentially expressed gene annotation and functional categorization

A cutoff value of 2-fold change (i.e. the absolute value of log_2_ ≥ 1) was adopted to identify genes that were differentially expressed and considered as potential candidate genes involved in the response to treatments. Gene Ontology (GO) analysis for gene information annotation and functional category distribution frequency was performed using the ErmineJ (version 3.0.2) software (max class size = 1500, min class size = 5) [98]. GO terms were assigned according to the gene ontology (www.go_daily-termdb.rdf.xml.gz) and the DBgrapego gene annotation analysis (http://www.genoscope.cns.fr/externe/Download/Projets/Projet_ML/data/8X/annotation/prot2go.filtered).

### Quantification of gene expression by quantitative RT- PCR (qRT-PCR)

For qRT-PCR quantitation of gene transcripts upon PHC treatment, grapevine canes were pruned to the 8^th^ dormant buds from the base and the 8^th^ dormant buds were immediately treated with 1 % (w/v) HC. Total RNA was extracted at 0, 6, 12, 24, 48 h post-treatment. For each time point, three replicates of bud samples were harvested for RNA extraction. First strand cDNAs was synthesized from 2 μg of total RNA using High Capacity cDNA Reverse Transcription Kit (P/N4368814, ABI, USA), according to the manufacturer’s instruction. Based on the results of microarray analysis, twelve grape genes, including five, six and one genes coding for ROS-generating, ROS-scavenging and NO-scavenging enzymes, respectively, were selected for quantification of gene expression by qRT-PCR. The forward and reverse primers for candidate and reference genes (Additional file 4) were designed using the primer Express® Software v2.0 (ABI, USA). A set of primers was designed for the eukaryotic initiation factor (*eIF4A*, accession #CO168508) [64], which served as a reference gene. qRT-PCR was performed using ABI PRISM 7500 Sequence Detection System (ABI, USA). One μL of synthesized cDNA was diluted five folds with water and used as template. Each assay was replicated three times. The amplification of target genes was monitored every cycle by SYBR-Green fluorescence (ABI, USA). PCR amplification begun with an initial denaturing step at 95 °C for 10 min, followed by 40 cycles of amplification at 95 °C for 15 s, 60 °C for 60s, 1 cycle of 95 °C for 15 s, 60 °C for 60 s, 95 °C for 15 s and 60 °C for 15 s.

## Additional files

Additional file 1: Changes in transcript abundance and clustering of significant differentially expressed genes. Changes in transcript abundance (M/A plot) from the top to bottom, relative mRNA abundance in P (A), HC (B) and PHC (C). The Y- and X-axis show M (log_2_ (Normalized Expressions Ratio) (by rank consistency-filtering LOWESS method) and A (log_2_ (gBGSubSignal x rBGSubSignal)^1/2^, representing the average signal of the two channels for each gene) obtained from microarray data, respectively. (DOCX 109 kb) Additional file 2: 239 up- and 106 down-regulated genes that were common regulated by P, HC and PHC treatments. (XLSX 34 kb) Additional file 3: Gene expression levels in ROS-related GO categories. The expression levels of significantly up-/down-regulated genes in the categories of ROS-generating, ROS-scavenging, ROS-specific transcription factors and NO-scavenging in grapevine buds treated with P, HC and PHC for 24 h, as revealed by ErmineJ analysis. (XLSX 20 kb) Additional file 4: List of primers used for qRT-PCR. (DOCX 16 kb)

## Abbreviations

ABI5: Abscisic insensitive
ADH: Alcohol dehydrogenase
AOX: Alternative oxidase
APX: Ascorbate peroxidase
Carboxy-PTIO: 2-(4-carboxyphenyl)-4,4,5,5-tetramethylimidazoline-1-oxyl-3-oxide potassium salt
CAT: Catalase
DAF-2DA: 4,5-diaminonaphthalene
DCF-DA: 2’,7́-dichlorofluorescin diacetate
DEGs: Differentially expressed genes
DHAR: Dehydroascorbate reductase
DHE: Dihydroethidium
ERF: Ethylene responsive factor
FAA: Formalin-acetic acid-alcohol
Fe-XO: Ferric-xylenol orange
GO: Gene ontology
GPX: Guaiacol peroxidase
GR: Glutathione reductase
GST: Glutathione S-transferase
HCN: Hydrogen cyanamide
KCN: Potassium cyanide
LOXs: Lipoxygenases
MDHAR: Monodehydroascorbate reductase
NO: Nitric oxide
NOS: Nitric oxide synthase-like
NOXs: NADPH oxidases
OCT: Optimal cutting temperature
P: Pruning
PBS: Phosphate-buffered saline
PCD: Program cell death
PDC: Pyruvate decarboxylase
PHCN: Pruning plus hydrogen cyanamide
qRT-PCR: quantitative RT-PCR
RBOHs: Respiratory burst oxidase homologues
RNS: Reactive nitrogen species
ROS: Reactive oxygen species
SNP: Sodium nitroprusside
SODs: Superoxide dismutases
SuSy: Sucrose synthase
TFs: Transcription factors
Trxh: Thioredoxin h
VvAOX: Vitis vinifera alternative oxidase 2
VvAPX3: Vitis vinifera ascorbate peroxidase 3
VvCAT1: Vitis vinifera catalase isozyme 1-like
VvDOX1: Vitis vinifera alpha-dioxygenase
VvERF: Vitis vinifera ethylene response element transcription factor
VvFSD: Vitis vinifera superoxide dismutase
VvFSD3: Vitis vinifera superoxide dismutase 3
VvGPX2: Vitis vinifera glutathione peroxidase 2
VvHB2: Vitis vinifera hemoglobin-2
VvHSF: Vitis vinifera heat stress transcription factor
VvPOD12: Vitis vinifera peroxidase 12
VvPOD72: Vitis vinifera peroxidase 72
VvRBOHA: Vitis vinifera respiratory burst oxidase homolog protein A
VvRBOHE: Vitis vinifera respiratory burst oxidase homolog protein E
VvWRKY: Vitis vinifera WRKY transcription factor
