# Supplementary material for: Hydrogen cyanamide breaks grapevine bud dormancy in the summer through transient activation of gene expression and accumulation of reactive oxygen and nitrogen species
Source: BMC Plant Biol. 2016 Sep 15;16:202. doi: 10.1186/s12870-016-0889-y (PMC5024461; doi:10.1186/s12870-016-0889-y)
Supplement: Additional file 1: — Changes in transcript abundance and clustering of significant differentially expressed genes. Changes in transcript abundance (M/A plot) from the top to bottom, relative mRNA abundance in P (A), HC (B) and PHC (C). The Y- and X-axis show M (log2 (Normalized Expressions Ratio) (by rank consistency-filtering LOWESS method) and A (log2 (gBGSubSignal x rBGSubSignal)1/2, representing the average signal of the two channels for each gene) obtained from microarray data, respectively. (DOCX 109 kb) [file 12870_2016_889_MOESM1_ESM.docx]

**Additional file 1. Changes in transcript abundance and clustering of significant differentially expressed genes.**

Changes in transcript abundance (M/A plot) from the top to bottom, relative mRNA abundance in P (A), HC (B) and PHC (C). The Y- and X-axis show M (log_2_ (Normalized Expression Ratio) (by rank-consistency-filtering LOWESS method) and A (log_2_ (gBGSubSignal x rBGSubSignal)^1/2^, representing the average signal of the two channels for each gene) obtained from microarray data, respectively.


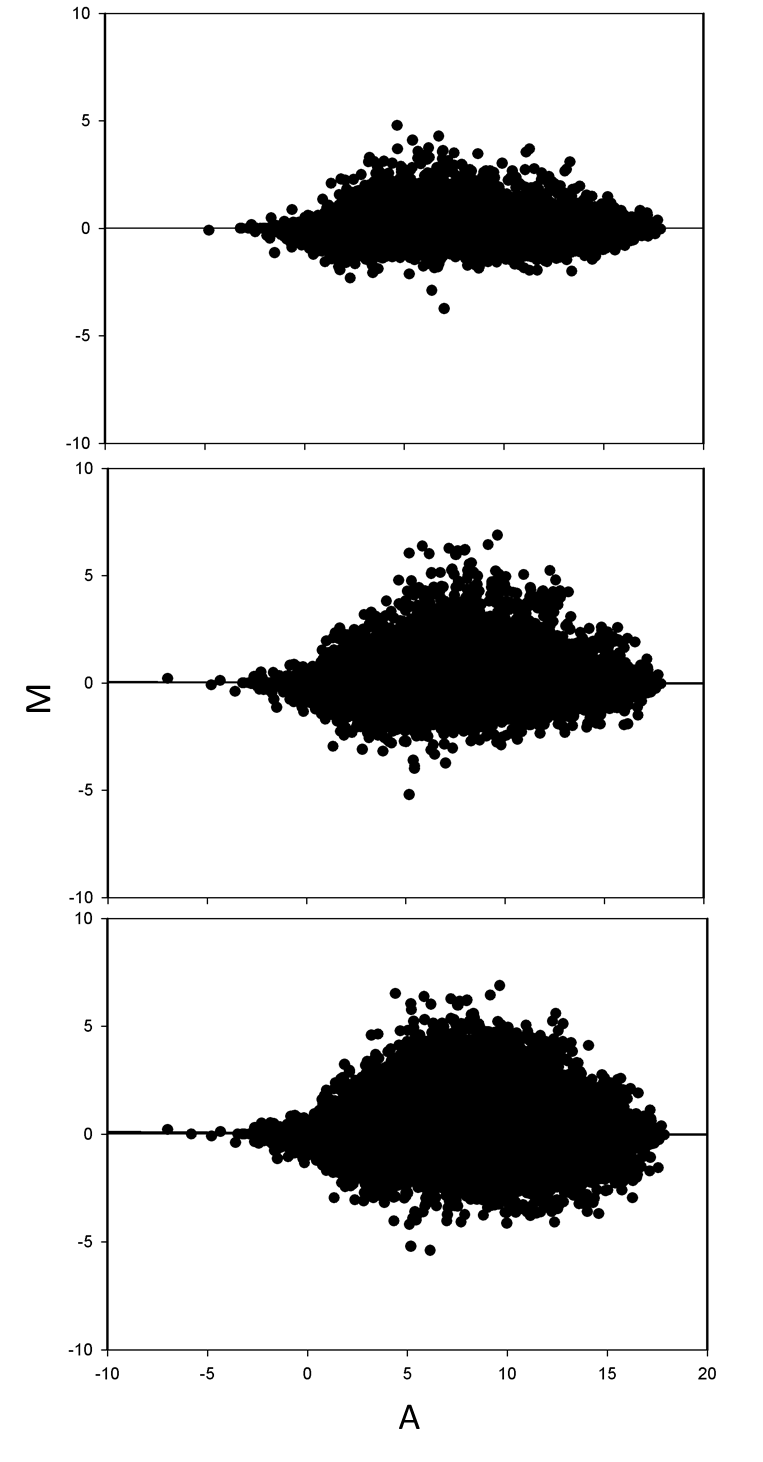


(C) PHC

(B) HC

(A) P
