# Supplementary material for: Hydrogen cyanamide breaks grapevine bud dormancy in the summer through transient activation of gene expression and accumulation of reactive oxygen and nitrogen species
Source: BMC Plant Biol. 2016 Sep 15;16:202. doi: 10.1186/s12870-016-0889-y (PMC5024461; doi:10.1186/s12870-016-0889-y)
Supplement: Additional file 4: — List of primers used for qRT-PCR. (DOCX 16 kb) [file 12870_2016_889_MOESM4_ESM.docx]

**Additional file 4: List of primers used for qRT-PCR.**

| **Gene Locus ID** | **Gene description** | **Primers** |
| --- | --- | --- |
| 1. *GSVIVT0001638600* | Vitis vinifera respiratory burst oxidase homolog protein E (VvRBOHE) | F: ATTATAGCCGCACGTACGATTTC  R: CTCCCCCGACGGCATAGT |
| 2. *GSVIVT00002525001* | Vitis vinifera respiratory burst oxidase homolog protein A (VvRBOHA) | F: CCCCCCAGTTCCGTTACAA  R: GGAGAAACTGCAGGGCATTG |
| 3. *GSVIVT00031598001* | Vitis vinifera peroxidase 72 (VvPOD72) | F: TGCCCCTGGCCTGCTT  R: GCATCACAGCCCCTGACAA |
| 4. *GSVIVT00031723001* | Vitis vinifera peroxidase 12 (VvPOD12) | F: CGCCGCTGGCTTGCT  R: GGCATCACATCCCTGAACAAA |
| 5. *GSVIVT00020782001* | Vitis vinifera apha-dioxygenase 1 (VvDOX1) | F: ATGCGCGCCAATTGGT  R: GCCCAAATGTGTCCTTGAATTT |
| 6. *GSVIVT00001920001* | Vitis vinifera glutathione peroxidase 2 (VvGPX2) | F: GCATTTCCTTGCAACCAGTTTC  R: GCAGCTTCCAGAATTTCCTCAT |
| 7. *GSVIVT00004080001* | Vitis vinifera catalase isozyme 1-like (VvCAT1) | F: GGACAGGCAAGAGCGTTTCA  R: GGTGACCCTTGGATCAGACAA |
| 8. *GSVIVT00036747001* | Vitis vinifera cytosolic ascorbate peroxidase (VvAPX3) | F: GAGCCCTGGAAGTTCGACAA  R: CAATCGCTTGGTTGATGATTTC |
| 9. *GSVIVT00007083001* | Vitis vinifera superoxide dismutase [Fe] 3, chloroplastic (VvFSD3) | F: TGCCCCAAGCTCCAAAATC  R: TGAAGCTTTTTGTGATCCATGAA |
| 10. *GSVIVT00014163001* | Vitis vinifera superoxide dismutase [Fe], chloroplastic (VvFSD) | F: GCTCAAGCCTCCTCCATATCC  R: CCAACGTCTCACGGCTCAT |
| 11. *GSVIVT00003173001* | Vitis vinifera alternative oxidase 2, mitochondrial (VvAOX2) | F: GACGGGCTCGGAAAATGG  R: GGCACGCCCCAGTAGCT |
| 12. *GSVIVT00036443001* | Vitis vinifera hemoglobin-2 (VvHB2) | F: GGCTGTTTTTGTTATGACTTGTGAA  R: CTCACTGTCACTTTGCCAGCTT |
| 13. *eIF4A* | Eukaryotic translation initiation factor 4A (reference gene) | F: ATGAACTTGCGCGAGGAGTT  R: GACCCCTCACACAGGGAATG |
